# Retinal Neurovascular Coupling in Diabetes

**DOI:** 10.3390/jcm9092829

**Published:** 2020-09-01

**Authors:** Garhöfer Gerhard, Jacqueline Chua, Bingyao Tan, Damon Wong, Doreen Schmidl, Leopold Schmetterer

**Affiliations:** 1 Department of Clinical Pharmacology, Medical University Vienna, 1090 Vienna, Austria; gerhard.garhoefer@meduniwien.ac.at (G.G.); doreen.schmidl@meduniwien.ac.at (D.S.); 2 Singapore Eye Research Institute, Singapore National Eye Centre, Singapore 169856, Singapore; jacqueline.chua.y.m@seri.com.sg (J.C.); bingyao.tan@ntu.edu.sg (B.T.); damon.wong@ntu.edu.sg (D.W.); 3 Academic Clinical Program, Duke-NUS Medical School, Singapore 169857, Singapore; 4 SERI-NTU Advanced Ocular Engineering (STANCE), Singapore 639798, Singapore; 5 Institute for Health Technologies, Nanyang Technological University, Singapore 308232, Singapore; 6 Center for Medical Physics and Biomedical Engineering, Medical University Vienna, 1090 Vienna, Austria; 7 Institute of Molecular and Clinical Ophthalmology, CH-4031 Basel, Switzerland

**Keywords:** neurovascular coupling, diabetes, retina, ocular blood flow regulation, functional hyperemia

## Abstract

Neurovascular coupling, also termed functional hyperemia, is one of the physiological key mechanisms to adjust blood flow in a neural tissue in response to functional activity. In the retina, increased neural activity, such as that induced by visual stimulation, leads to the dilatation of retinal arterioles, which is accompanied by an immediate increase in retinal and optic nerve head blood flow. According to the current scientific view, functional hyperemia ensures the adequate supply of nutrients and metabolites in response to the increased metabolic demand of the neural tissue. Although the molecular mechanisms behind neurovascular coupling are not yet fully elucidated, there is compelling evidence that this regulation is impaired in a wide variety of neurodegenerative and vascular diseases. In particular, it has been shown that the breakdown of the functional hyperemic response is an early event in patients with diabetes. There is compelling evidence that alterations in neurovascular coupling precede visible signs of diabetic retinopathy. Based on these observations, it has been hypothesized that a breakdown of functional hyperemia may contribute to the retinal complications of diabetes such as diabetic retinopathy or macular edema. The present review summarizes the current evidence of impaired neurovascular coupling in patients with diabetes. In this context, the molecular mechanisms of functional hyperemia in health and disease will be covered. Finally, we will also discuss how neurovascular coupling may in future be used to monitor disease progression or risk stratification.

## 1. Introduction

Diabetes is a complex systemic disease, frequently accompanied by serious long-term microvascular and macrovascular complications [1]. These complications affect a wide variety of different organs in the human body, including the eye. As such, diabetic eye diseases—more importantly diabetic retinopathy and diabetic macula edema—are among the most sight-threatening ocular pathologies worldwide, and their prevalence is expected to considerably increase in the near future [2,3].

Although the pathogenesis of diabetic retinopathy has not yet been fully elucidated [4], there is compelling evidence that vascular changes, ocular perfusion disturbances as well as tissue hypoxia play a major role in its pathogenesis [5,6]. Impaired blood flow may cause under- or overperfusion, which in turn triggers microvascular complications [7]. Blood flow in the eye cannot be considered to be static but is dynamically regulated in response to various stimuli [8]. More specifically, as discussed in detail below, ocular blood flow is adapted in response to exogenous factors such as changes in ocular perfusion pressure [9] (usually referred to as pressure autoregulation) or to metabolic stimuli such as hypoxia or hyperoxia [10,11]. In addition, tissues with high neural activity such as the retina show an intrinsic mechanism to adapt blood flow to increased metabolic demands caused by firing neurons. This regulatory response, which is called functional hyperemia or neurovascular coupling, is a key element that ensures the supply of the retinal tissue with oxygen and other nutrients to changing local metabolic requirements due to changing neural activity [12]. As discussed in detail below, an intact neurovascular coupling mechanism is highly dependent on a complex interaction between neurons and blood vessels to meet the metabolic demand that arises from increased neural activation. Malfunction of neurovascular coupling may lead to transient hypoperfusion and trigger other detrimental consequences such as local hypoxia and cellular damage [13,14].

The current review focuses on retinal functional hyperemia in patients with diabetes. We will discuss the role of neurovascular coupling as an intrinsic mechanism to adapt perfusion to changes in metabolic demand and how an impairment of this regulation process may contribute to the pathogenesis of diabetic complications in the eye. Further, we will describe how neurovascular coupling may in the future be used to monitor disease progression or risk stratification.

## 2. Ocular Vasculature and Diabetes

The human eye is the only organ in the human body where direct and non-invasive visualization of the microcirculation is possible. Briefly, the posterior part of the eye receives its blood supply through two different and independent vascular beds: the retinal circulation, which nourishes the inner part of the retina, and the choroid, supplying the outer part of the retina including the photoreceptors. The central retinal artery enters the eye with the optic nerve head and then branches into an upper and lower part, which in turn divides into a temporal and nasal vessel branch. When entering the optic nerve head, the central retinal artery has a diameter of approximately 170 µm [15]. Whereas fundus-camera-based systems allow for the measurements of vessel calibers down to approximately 60 µm, which corresponds to the caliber of peripheral branch arteries, more sophisticated techniques allow for visualization of retinal vessels down to the capillary level [16]. Thus, researchers were interested for decades in identifying microvascular changes at the level of the retina and in determining whether they can provide information regarding systemic cardiovascular diseases such as arterial hypertension, arteriosclerosis, or stroke [17,18,19]. 

It has been speculated that changes in retinal vessel calibers may serve as biomarkers for the prediction of cardiovascular events such as myocardial infarction or stroke and allow for the early identification of high-risk patients [20].

In diabetes, microvascular complications, where retinal neovascularization or microvascular lesions are predominantly seen, it does not come as a surprise that the role of ocular blood flow and its regulation has gained considerable interest. Indeed, it is generally accepted now that retinal vessels are early and common targets of diabetic damage and might serve as biomarkers for diabetes-related microvascular complications [21].

**Figure 1:**
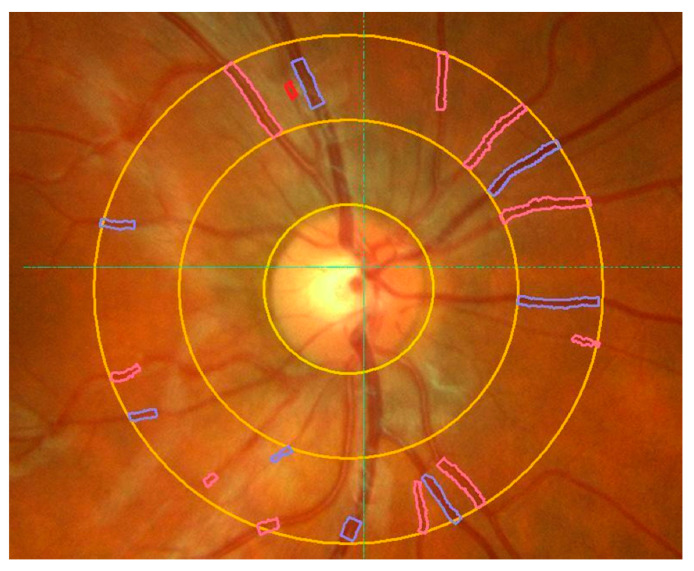
Automatic assessment of retinal vessel diameters using a commercially available retinal vessel analyzer. Retinal vessels enter the eye via the optic nerve head (ONH) and then divide into branches (see text for details). Vessel calibers of retinal arteries (red) and retinal veins (blue) are automatically detected by the software. Yellow circles indicate the ONH and one and two disc diameter distances from the ONH. The arterio-venous ratio (A-V) ratio is calculated automatically by the software.

Starting in the 1990s, computer-assisted analysis methods for precise in vivo assessment of retinal vessel calibers based on digitized fundus photographs became available allowing for the non-invasive quantification of retinal vessel diameter changes in large-scale studies [22,23]. More recent fundus-camera-based systems extend this approach and allow for the automatic assessment of several more complex vessel-related measures such as the arterio-venous ratio (Figure 1).

Using this approach, a variety of epidemiological studies have investigated alterations in retinal vessel diameters in patients with diabetes and their potential use as a biomarker for the progression of the disease. The studies, however, produced mixed results. Prospective data from two large epidemiological studies, the Atherosclerosis Risk in Communities (ARIC) Study and Beaver Dam Eye Study, showed that non-diabetic individuals with a smaller arterio-venous ratio, a measure based on the ratio of total retinal arterial diameter to that of retinal veins, had a considerably higher risk of incident diabetes [24,25]. A comparable result was found in the Australian Diabetes, Obesity, and Lifestyle Study, which reported that retinal arteriolar narrowing predicts the incidence of diabetes [26]. 

In contrast to these findings, other studies found increased retinal vessel calibers [27] or failed to identify a clear association between vessel calibers and incident diabetes [28,29]. The reason for these differing results is unclear but may be at least partially related to differences in the methodology used for vessel caliber measurement. Most of the large epidemiological studies have used the ratio between retinal arterial and venous diameters to assess changes in retinal vasculature independent from the individual angio-architecture and the refraction of the eye [23]. This method largely relies on the assumption that venous vessel diameters are not affected by the disease. This might, however, not necessarily be the case: The authors of the Rotterdam Eye Study state that the changes in arterio-venous ratio as observed in some of the studies may be attributed rather to venous dilatation than to arteriolar constriction as previously hypothesized [29]. The latter interpretation is also strengthened by a recent meta-analysis including data of more than 18,000 individuals, which reports that wider retinal venules but not narrower retinal arterioles are associated with an increased risk for diabetes [30]. 

In addition to the disease per se, vessel calibers may be sensitive to changes in glucose concentrations, which further complicates the interpretation of the results [31]. For a more in-depth description and discussion regarding retinal vessel diameters as a potential biomarker for diabetes, the reader is referred to review articles published previously [21,32,33].

## 3. Microvascular Changes in Patients with Diabetes

**Figure 2:**
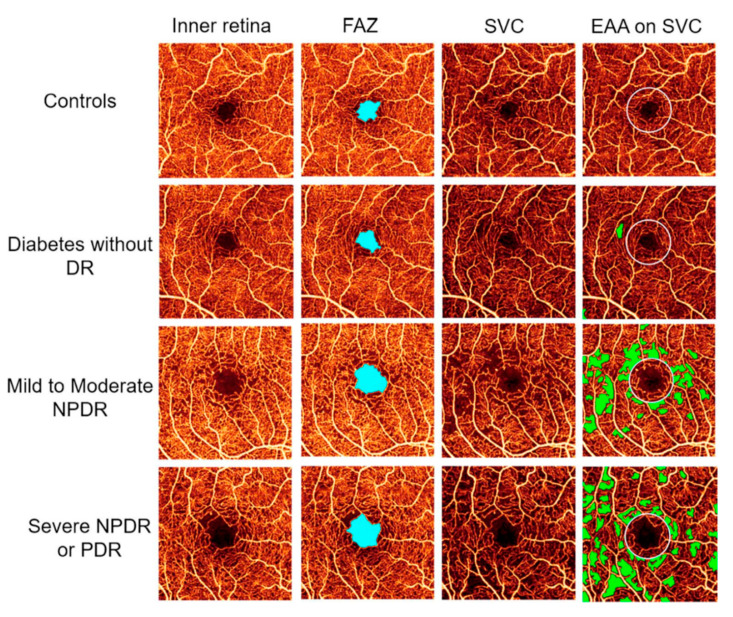
Optical coherence tomography angiography of the inner central retina in healthy subjects and in patients with diabetic retinopathy of different severities (first column). Second column shows the foveal avascular zone (FAZ, light blue), third column the superficial vascular layer (SVC), and the fourth column the extrafoveal avascular area (EAA, green) on the SVC. From [45] with permission.

It is well established that microvascular changes are an early event in the disease process [1]. Among these, microaneurysms are the earliest detectible alterations in diabetic retinopathy [34]. Clinically, microaneurysms can be identified by ophthalmoscopy as deep-red dots varying from 25 to 100 µm in diameter. There is general agreement that the presence and number of microaneurysms have strong predictive value with respect to progression of diabetic retinopathy [35]. Further anatomical alterations such as thickening of the vascular basement membrane and pericyte loss are also well described as early pathological features in patients with diabetes [7]. Recent technical advances in ocular imaging techniques, most importantly the introduction of optical coherence tomography angiography (OCT-A), allow for the non-invasive visualization and quantification of retinal microvascular changes [36]. OCT-A provides depth-resolved images of the retinal and choroidal vasculature approaching almost histological resolution, which has considerably increased our understanding of microvascular alterations in patients with diabetes [37]. Furthermore, OCT-A allows for the quantification of vascular density in different layers of the retinal vascular tissue and might, therefore, be a suitable tool to monitor disease progression. Using this technology, it has been shown that patients with diabetes exhibit an increase in the foveal avascular zone [38,39,40] most probably due to capillary loss and/or a decrease of parafoveal blood flow (Figure 2). In addition, it is reported that retinal vessel density as measured using OCT-A is reduced in patients with diabetes depending on the severity of the retinopathy [41,42,43,44,45,46]. The reader is referred to a recent review article for a more in-depth discussion regarding the OCT-A findings in persons with diabetes [47].

## 4. Diabetes and Blood Flow

Although the above-mentioned results indicate that patients with diabetes have altered ocular perfusion, diameter measurements alone are insufficient to draw conclusions on volumetric blood flow. To investigate whether ocular perfusion is compromised in patients with diabetes, more sophisticated measurement techniques, which allow for the assessment for blood flow, are necessary. However, currently no gold standard for the measurement of retinal blood flow exists and the available methods are technically demanding or suffer from limitations that limit their use in patients with ocular diseases [48,49].

The current evidence regarding blood flow changes in patients with diabetes is sparse and sometimes contradicting. Although most of the studies found changes in the blood flow in patients with diabetes, there is still no consensus regarding the pattern of blood flow changes with respect to different types of diabetes or the clinical severity of the disease. More specifically, both reduced [50,51,52] and increased blood flow [53,54,55,56] have been reported in the retina of diabetic patients, depending on the type of diabetes and the method used for blood flow measurement.

As stated above, a wide variety of methods have been used to study retinal blood flow, and the techniques suffer from methodological problems, including poor reproducibility [48]. Doppler optical coherence tomography may in future overcome these limitations, but currently only prototype commercial systems are available [57]. In addition, the majority of the published studies were of small sample size and mostly cross sectional, limiting the interpretation and the general applicability of the results. To truly characterize retinal perfusion in patients with diabetes, studies in larger cohorts as well as longitudinal trials are warranted.

## 5. Regulation of Retinal Blood Flow

It is known for decades that the human retina is a metabolically demanding tissue. Thus, small alterations in blood flow and/or oxygen or shortage of nutrients may lead to an impaired supply of the tissue and, in turn, to hypoxia and a subsequent risk of tissue damage [58]. Conversely, exposure of tissue to hyperperfusion and/or high oxygen tension may lead to an increased formation of reactive oxygen species again followed by tissue dysfunction and cell death [59]. As such, it does not come as a surprise that ocular blood flow is highly regulated in response to changes in ocular perfusion pressure and metabolic factors [9,60,61]. For example, changes in systemic blood pressure or intraocular pressure [62] lead to an autoregulatory response of the ocular vasculature, thereby adapting vascular resistance in order to keep blood flow constant [61].

Metabolic regulation of retinal blood flow has also been reported, and vascular tone is sensitive to changes in O_2_ and/or CO_2_: The retinal circulation adapts its blood flow to changes in blood O_2_ and CO_2_ levels to compensate for changes in blood gas concentrations. High O_2_ levels lead to a pronounced decrease in retinal vessel diameter and blood flow in order to prevent excessive oxygen supply to the retina [11,63]. Hypercapnia leads to vasodilation and an increased perfusion of the retina [64], an effect that is also well established for the brain. Retinal blood flow is, however, not only regulated in response to changes in perfusion pressure and oxygen but also in dependence on local neural activity, which is usually referred to as neurovascular coupling or functional hyperemia.

## 6. The Concept of Neurovascular Coupling

The first description that blood flow in neuronal tissues can be adapted to changes in local metabolic demands comes from the brain. In their landmark paper from 1890, Roy and Sherrington have hypothesized that the brain possesses an intrinsic mechanism that allows for the adaptation of blood flow to changes in neural activity with high spatial and temporal resolution [65]. This coupling mechanism between neural activity and blood flow is referred to as functional hyperemia or neurovascular coupling [66,67]. According to the current scientific view, functional hyperemia ensures adequate supply of the working tissue with nutrients and oxygen to cover the local energy demand of the firing neurons in correspondence to their functional activity.

Today, most of the evidence regarding the role of neurovascular coupling in health and disease stems from brain research. Modern functional brain imaging capabilities such as positron emission tomography (PET) or functional resonance imaging [68] have proven the theory of neurovascular coupling by showing that increased neural activity in the brain is accompanied by a pronounced and highly localized increase of local blood flow [69,70,71].

**Figure 3:**
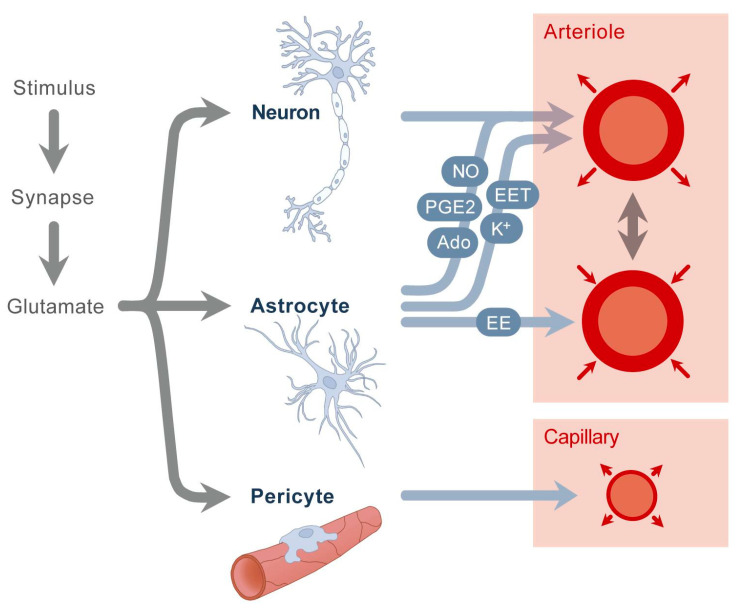
Neurovascular unit. A stimulus causes increased workload of neurons, which triggers a cascade of different events. Glutamate stimulates neurons and astrocytes, releasing nitric oxide (NO), potassium (K+), adenosine (Ado), epoxyeicosatrienoic acids (EETs), and prostaglandins (PG E_2_). Astrocytes also secrete arachidonic acid (EE), which causes vasoconstriction. A second mechanism is the stimulation of pericytes, resulting in capillary vasodilation. Modified from [73] with permission.

However, despite more than 25 years of intensive research, the cellular mechanisms mediating the coupling between neural activity and blood flow in the brain are not fully understood and remain controversial. It has recently become clear that the process of functional hyperemia requires a highly complex communication between different cell types and tissues involving neurons, glia cells, and blood vessels, with glia cells playing a central role in the signal pathway [67]. This complex regulation system ending up in the pericytes and the vascular smooth muscle cells is usually referred to as the “neurovascular unit” (Figure 3) [72].

In the past, both metabolic feedback mechanisms as well as activity-dependent feed-forward mechanisms have been differentially discussed as potential pathways to mediate functional hyperemia in the brain. As such, activity-induced local increases in CO_2_, proton concentrations, or the release of lactate has been proposed as a metabolic trigger for the release of vasoactive agents [74,75]. However, whether and to what extent these metabolic changes contribute to the regulation processes warrants further investigation. Alternatively, activity-induced release of neurotransmitters by firing neurons has been hypothesized to activate blood flow. According to this so-called feed-forward mechanism, neuromodulatory agents such as K^+^ and/or others may initiate a signaling network including nitric oxide (NO), prostanoids, and others that regulate vascular tone in dependence on neural work load [67,76]. 

It has also been hypothesized that several parallel signaling pathways need to be involved in mediating functional hyperemia in the brain to maintain energy balance. This ensures that the brain can compensate for an impaired pathway by recruiting other alternative signaling cascades. An in-depth discussion of the exact molecular mechanism behind this regulation process in the brain is beyond the scope of this article, and the reader is referred to recent review articles [67,77,78]. 

### 6.1. Neurovascular Coupling in the Retina

As the retina is a tissue of neural origin, it does not come as a surprise that neurovascular coupling can also be observed in the retina [12,79]. These similarities also apply to the molecular mechanisms that underly functional hyperemia at the level of the retinal microcirculation. Experimental evidence from rodent animal models indicates that neurovascular coupling in the retina is also largely mediated by glia cells [80,81]. Müller cells and astrocytes are the principal glia cells of the retina and optic nerve head. When stimulated, they release vasoactive agents such as prostaglandins (PG), epoxyeicosatrienoic acids, and 20-hydroxy-eicosatetraenoic acids, leading to vasodilatation [80]. Along this line of thought, it has been shown that interruption of signaling from neurons to glial cells reduces the flicker-induced hyperemic response [80]. 

Nitric oxide (NO), a potent vasodilator, has been shown to play a modulatory role in the mediation of functional hyperemia. Early experiments using invasive techniques for the assessment of NO in animal studies revealed an increase in local NO concentration during photic stimulation [82,83]. Blocking NO-synthase by NG-monomethyl-L-arginine significantly reduces flicker-induced vasodilatation in both animals and humans [84]. More recent animal studies indicate that NO does not directly mediate flicker-induced hyperemia but exerts a complex modulatory function [80]. NO interacts with glia cells and is involved in the regulation of the release of vasodilatation agents, most importantly PG E_2_ [85]. A detailed summary of the current understanding of the cellular mechanisms underlying neurovascular coupling in the retina has been published [12,86,87].

### 6.2. Neurovascular Coupling and Retinal Blood Flow

It is generally accepted that increased retinal neural activity induced by photic stimulation, i.e., with flickering light, is associated with a pronounced and immediate hyperemic response. Experimental evidence indicates that this functional hyperemia can be observed in both the vascular bed of the optic nerve head as well as in the retinal circulation. Numerous studies using laser Doppler flowmetry (LDF) to assess optic nerve head perfusion consistently report that blood flow almost doubles in response to stimulation with flicker light in both experimental animal models [88] and humans [89].

**Figure 4:**
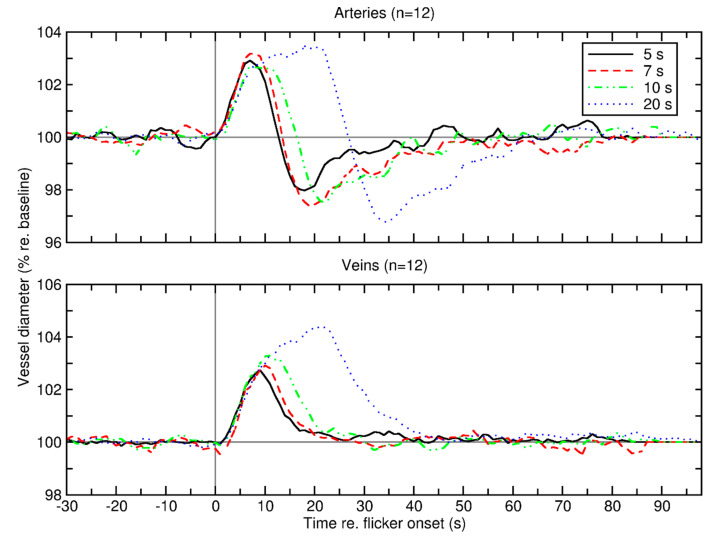
Average retinal vessel diameter of 12 healthy subjects before, during, and after secession of retinal vessel diameter for arteries (top panel) and veins (bottom panel). Different colors represent 4 different lengths of stimulation duration (5, 7, 10, and 20 s). Vertical gray line denotes flicker onset; horizontal gray line denotes baseline diameter normalized to 100%. Reproduced from [93] with permission.

The effect of photic stimulation on retinal vessel calibers is also well described. Studies investigating neurovascular coupling in both animals and humans report that stimulation with flickering light is accompanied by a vasodilation of retinal vessels in the range of approximately 3–5% [90,91,92]. Interestingly, this increase in vessel calibers consistently occurs very rapidly after the onset of photic stimulation [93]. More specifically, the flicker profile follows a specific distinct pattern: For retinal arteries, a rapid vasodilation, which mostly occurs within the first 5–10 s of light stimulation is followed by a maintenance phase where the diameter is mostly stable (Figure 4). Upon cessation of the flicker stimulation, diameters rapidly drop below baseline before slowly returning back to the original values [93,94]. In contrast to retinal arteries, retinal veins lack a transient vasoconstrictor response and slowly return back to baseline values [93]. The reason for this behavior is unclear but may be related to different regulatory mechanism with different time-courses involved in the hyperemic response of retinal vessels.

Interestingly, the hyperemic response to flicker stimulation seems to be rather stable and is largely independent of changes in intraocular pressure or ocular perfusion pressure. Experimental evidence shows that a short-term increase in intraocular pressure of up to approximately 40 mmHg does not block or alter the flicker-induced hyperemia in either the retina or the optic nerve head [95]. Similarly, acute increases of perfusion pressure, such as for example that induced by isometric exercise, do not induce a major effect on the flicker-induced vasodilatation in humans [96]. 

Additionally, the flicker response in both the retinal circulation as well as the optic nerve head is independent of stimulation frequency and modulation depth of the stimulus within a relatively wide range. Although studies that systemically investigate the influence of different stimulation patterns and modulation depths are sparse, early experimental data from feline animal models indicate a band pass shape of flicker responses with an almost stable optic nerve head blood flow response in the frequency range between 5Hz and 20Hz [97,98]. Accordingly, the same studies report that flicker response is rather stable over large ranges of modulation depths, starting at approximately 10% of modulation depth with a saturation at 50% [97,98]. Since the latter results closely resemble the functional responses of retinal ganglion cells, the findings support the hypothesis that flicker-induced hyperemia is mainly dependent on increased retinal ganglion activity [12]. 

As for human experiments, the vast majority of studies published in the literature used either square-wave-modulated flicker lights or flashlight stimulations to investigate flicker-induced hyperemia. Given that the influence of modulation depth, wavelength, and frequency on flicker response has not been thoroughly investigated, there is currently no certainty regarding the optimal stimulation pattern in humans. Polak and colleagues have investigated the influence of flicker frequency on flicker-induced changes of retinal vessel diameter in humans. The authors report that in all tested flicker frequencies between 2 and 64 Hz flicker-induced vasodilatations were observed with a more pronounced response in retinal arteries compared to that in retinal veins [94]. However, in this study, short-wavelength chromatic flicker was used to separate stimulation light from the illumination pathway of the measuring system. Results indicate that both parvo- and magnocellular neural pathways are activated during flicker stimulation, which highlights the importance of the selected wavelength range for visual stimulation [99]. 

Mainly because of technical restrictions, most of the previously mentioned studies have focused on the response of retinal vessel diameters to investigate neurovascular coupling and draw conclusions on flow changes. More sophisticated technical approaches allow, however, not only for the assessment of retinal vessel calibers but for the direct determination of blood flow changes during flicker stimulation. Assessing volumetric blood flow by a combination of Laser Doppler Velocimetry and retinal vessel analysis has shown that retinal blood flow in major retinal vessels increases by more than 50% during visual stimulation [100], which is slightly higher compared to the data obtained for the optic nerve head [89]. Given that this approach requires excellent cooperation of the subject and is very time demanding, this technique is not suitable for clinical use to investigate functional hyperemia in patients.

However, the rapid development in ocular imaging may in future enable us to overcome the latter limitations. As such, more recently, optical technologies have emerged that allow for the measurement of retinal perfusion based on functional optical coherence tomography (OCT). Using dual-beam bidirectional Doppler Fourier Domain OCT (D-OCT), it has been shown that total retinal blood flow increases by approximately 50% in response to flicker light stimulation, which is in line with results obtained using other methods [101]. The data of the latter study report individual increases in total retinal blood flow between 34% and 66% [101]. Although the reason for the high inter-subject variability is unclear, it may be related to the differences in the angio-architecture and the variability of total retinal blood flow per se [102]. However, as the D-OCT technique is not yet commercially available, its use is currently limited to specialized laboratories.

Another method that has recently gained interest as a non-invasive method to assess neurovascular coupling in humans is laser speckle flowgraphy (LSFG). As shown previously with other techniques, the LSFG technique allows the quantification of flicker-induced hyperemia in the retinal circulation and the optic nerve head [103]. This technique is also commercially available and may in future be used as a non-invasive, easy-to-use tool to assess neurovascular coupling in humans.

### 6.3. Neurovascular Coupling in Diabetes

Convincing data across studies show that neurovascular coupling is impaired in patients with diabetes. Using fMRI and other functional imaging techniques for the brain, there is compelling evidence that there is an uncoupling between neural activity and blood flow in brain microcirculation in patients with diabetes mellitus, which in turn may trigger clinical consequences such as the cognitive impairment frequently observed in patients with type II diabetes [104,105].

As for the retina, it has been shown that vascular dilatation evoked by flicker stimulation is reduced in both patients with type I and type II diabetes when compared to that in healthy, non-diabetic subjects [106,107,108,109]. Some of these studies indicate that alterations in flicker-induced vasodilatation is evident also in early stages of diabetes with no or minimal diabetic retinopathy [106,107]. 

**Figure 5:**
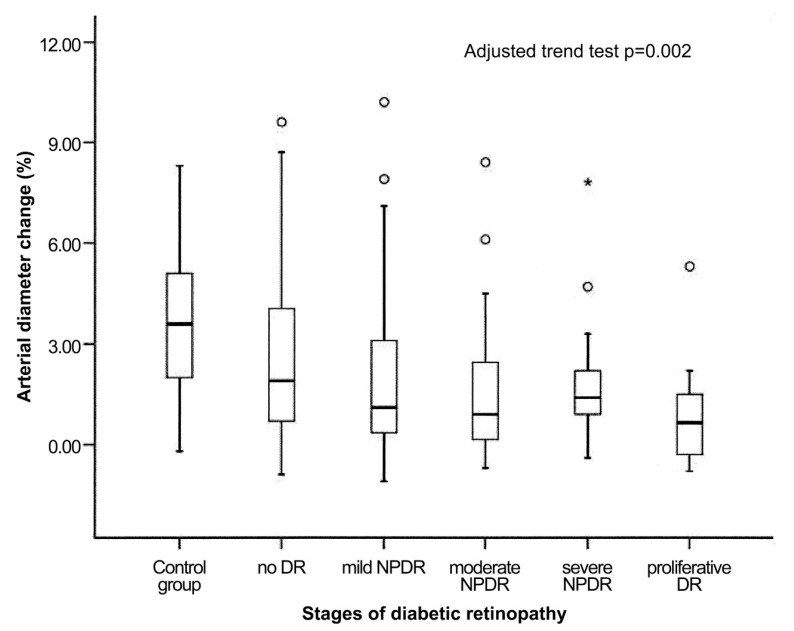
Flicker-induced vasodilatation of retinal arterial diameters as percent change from baseline in healthy subjects (control group) and different severities of diabetic retinopathy (DR = diabetic retinopathy, NPDR = non-proliferative diabetic retinopathy). Circles represent outliers and the asterisk represents an extreme outlier. From [110] with permission.

These results are also consistent with data from a larger trial, investigating a cohort of approximately 200 patients with type II diabetes [110] demonstrating that the flicker-induced vasodilatation of both retinal arteries and veins is abnormal even in patients without diabetic retinopathy, strongly suggesting that abnormal neurovascular coupling precedes a visible angiopathy in humans. Furthermore, the authors report that the flicker response deteriorates with increasing stages of diabetic retinopathy, which indicates that the impairment of functional hyperemia is dependent on the severity of the disease (Figure 5) [110]. 

In the previous study [110], no adjustment for potential confounding systemic factors such as smoking, fasting glucose, and blood pressure was performed. Factors such as insufficient glycemic control [111,112], chronic systemic hypertension [113] or hypercholesteremia [114] may, however, per se lead to an impaired flicker response. Taking into account these potentially biasing factors, data from a cross-sectional study including 224 individuals with diabetes and more than 100 healthy controls confirmed that functional hyperemia is reduced in patients with diabetes [115]. In particular, the authors showed that after adjustment for age, sex, diabetes duration, fasting glucose, cholesterol and triglyceride levels, current smoking status, systolic blood pressure, and use of antihypertensive and lipid-lowering medications, subjects with low flicker responses were more likely to have diabetes [115]. In addition, it is reported by the same group of authors that reduced flicker-induced vasodilation is associated with a wider retinal vascular caliber [116]. 

Given that diabetic retinopathy displays regional differences in respect to the occurrence of retinopathy lesions, it has been hypothesized that these differences may be caused by regional loss of the neurovascular coupling, in particular in the periphery of the retina [117].Indeed, cross-sectional studies report regional differences in flicker-induced hyperemia in patients with diabetic retinopathy [117] and patients with diabetic macular edema (DME) [118]. Although the data of the latter studies indicate that there is a generally diminished response in patients with proliferative diabetic retinopathy [117] and DME [118], the authors suggest that central retinal areas might be less affected by impaired blood flow regulation processes than more peripheral regions. Whether this hypothesis holds truth or whether the results are related to the peripheral dropout of vessels has yet to be determined.

In contrast to the large variety of studies mentioned above, one study did not find an attenuated flicker responses in patients with type 2 diabetes when compared to a healthy, age-matched control group [119]. However, this trial included 20 patients only and may, therefore, be underpowered to detect differences between healthy and diabetic subjects.

Retinal flicker-induced hyperemia has also been investigated in different diabetic animal models including rats and mice. These experiments show that in streptozotocin-induced rat models of type 1 diabetes flicker-induced hyperemia is reduced by more than 50%, which is well compatible with the results obtained in humans [81,85,120]. The loss of functional hyperemia can be reversed by the administration of inducible nitric oxide synthase (iNOS) inhibitors [81,85]. 

Although there is compelling evidence that flicker-induced hyperemia is reduced in subjects with diabetes, the molecular mechanisms behind this altered response are poorly understood. As stated previously, NO seems to have a central modulating role during functional hyperemia, most probably by regulating glial release of EETs and PGE_2_ [80]. Data from human studies suggest that NO may also play a role in the altered functional hyperemic response in patients with diabetes. In particular, an interventional study provides evidence that administration of the NOS inhibitor L-NMMA leads to an increase in flicker-induced vasodilation in patients with diabetic macula edema [118]. Whether this is related to an increased NO concentration at the level of the retina in patients with diabetes needs to be clarified in further studies.

Further, it has been suggested that the attenuation of flicker response found in patients with diabetes is consistent with endothelial dysfunction [110,115]. According to this hypothesis, the loss of the hyperemic response may be a direct consequence of impaired endothelium-dependent dilatation on the microvascular level. In patients with type I and type II diabetes, reduced endothelial-dependent vasodilatation was shown in several clinical studies using plethysmography of the brachial or femoral arteries [121,122].

Indeed, patients with known endothelial dysfunction, such as chronic smokers [123], those with hypercholesteremia [113] or patients with systemic hypertension [114] show reduced hyperemic responses to flicker stimulation. Interestingly, a study testing the general vasodilatation response of retinal vessels by the exogenous administration of glyceryl trinitrate, a potent endothelium-independent NO donor, revealed that the response of retinal vessels to NO is preserved in patients with type II diabetes [124]. This indicates that abnormal flicker-induced vasodilatation in diabetes is not a consequence of generally reduced retinal vascular reactivity but is due to compromised intrinsic mechanisms that promote functional hyperemia [124]. One study investigating a potential correlation between flicker-induced vasodilatation and other systemic measures of endothelial function such as flow-mediated vasodilatation (FMD), failed to show a strong correlation between FMD and flicker response [125]. Whether this is related to the different vascular beds measured or the stimulus used has yet to be investigated. 

Considering the latter findings, it has been hypothesized that a reduced “vasodilatory reservoir” or the inability of vessels to further dilate in already dilated retinal arterioles and venules in patients with diabetes causes a decrease in flicker response [126]. As stated above, this hypothesis is strengthened by the observation that venous dilatation is frequently observed in patients with diabetes. However, as vasodilatation induced by external nitric oxide is preserved in predilated vessels of patients with diabetes [124], it seems unlikely that reduced flicker responses can be solely attributed to different baseline vessel calibers. 

Finally, impaired neuronal function may contribute to the diminished flicker response found in patients with diabetes. It has been hypothesized that beside microvascular changes, neuroglial impairment may be involved early in the disease process [127]. Retinal oscillatory potentials, which are an early functional retinal abnormality in patients with diabetes, negatively correlate with retinal arteriolar caliber indicating a close relationship between neuronal microvasculature dysfunction in the retina [128]. Müller cells, which play an important role in mediating neural activity, are affected early in diabetes and exhibit different pathological properties such as the diminution of potassium channels and an impairment of intracellular fluid transportation [129]. Based on these observations, it is reasonable to suggest that impaired neural activity, reduced glia cell activation, and an impaired signal transduction contribute to reduced flicker-induced vasodilatation.

Along this line of thought, Lecleire-Collet and co-workers investigated a potential correlation between neural-activity and flicker-induced retinal vasodilation in patients with diabetes and healthy controls [107]. Although the authors found an overall correlation between flicker-light-induced arterial vasodilatation and pattern electroretinography (ERG) in the whole study group, no correlation was observed when only diabetic patients were considered. Another study investigated pattern electroretinography and flicker-induced vasodilatation in a group of 50 patients with type I diabetes without any signs of retinopathy and 50 age- and sex-matched healthy control subjects [130]. This study confirmed previous results of diminished flicker-induced vasodilation in patients with no visible diabetic retinopathy; no difference was found in terms of pattern ERG between groups. This indicates that the abnormal retinal hyperemic response is unlikely to be a consequence of reduced neuronal activity but rather precedes ERG changes. Further research investigating the role of neuroglia cells and their potential influence on neurovascular coupling is required. Additionally, longitudinal studies may add information regarding the time-course of neural and endothelial dysfunction in patients with diabetes.

### 6.4. Neurovascular Coupling in Other Diseases

As stated previously, a breakdown of neurovascular coupling is not specific to patients with diabetes. Several studies have shown that neurovascular coupling is dependent on age [131,132,133]. Using functional MRI, data from the brain indicate that neurovascular coupling blood flow responses are reduced in older adults compared to those in younger subjects [133]. This is in keeping with experimental evidence from rodent models showing a significant impairment of neurovascular coupling in animals with advanced age [134,135]. Thus, it has been hypothesized that a breakdown of neurovascular coupling may exacerbate cognitive decline during aging [66]. Interestingly, selective pharmacological blocking of neurovascular coupling in young healthy animals leads to a pronounced impairment of cognitive and sensorimotor function [136] whereas rescue of functional hyperemia is associated with significantly improved spatial working memory and learning [137]. This supports the hypothesis that an age-related impairment of neurovascular coupling in the brain plays a causal role in the pathogenesis of dementia.

For the retina, the data regarding the age dependence of neurovascular coupling is less clear. In a study investigating the effect of age, blood pressure, and baseline retinal vessel diameter, the authors did not find a correlation between age and flicker-induced vasodilation [113]. In contrast, investigating flicker-induced vasodilatation in the human retina, Lipecz and colleagues report that individuals with an age of 65 years or more have diminished flicker responses compared to a younger control group at the age of 45 or less [132]. Further studies are required to investigate the associate between aging and neurovascular coupling in the retina.

Additionally, it has been previously shown that a reduction of the functional hyperemic response of retinal vessels can also be observed in patients with other ocular diseases. As such, there is ample evidence that patients with glaucoma show a reduction of flicker-induced vasodilation [138,139,140,141]. In addition, it has been shown that a decline in flicker response is also present in patients with systemic vascular diseases such as systemic hypertension [113] or hypercholesterolemia [114]. Attempts have also been made to use neurovascular coupling as a potential biomarker for neurodegenerative diseases of the brain. Although one study found an increase in flicker response in patients with Alzheimer’s disease [142], another study failed to find a difference in flicker-induced hyperemia in patients with Alzheimer´s disease and mild cognitive impairment [143].

## 7. Further Studies

Most of the studies investigating neurovascular coupling in patients with diabetes are cross-sectional and have included only a limited number of participants. Although cross-sectional studies indicate stage-dependence of the reduction in functional hyperemia, longitudinal data from large, multicentric studies are currently lacking. This is also related to the fact that currently no gold standard for the measurement of retinal blood flow is available. The assessment of neurovascular coupling in humans is technically demanding and only available in specialized research centers. However, the rapid technical development of new functional imaging methods such as functional optical coherence tomography may, in future, allow for the easy and non-invasive assessment of volumetric blood flow also in clinical settings. 

Additionally, it is unclear whether patients with earlier or more pronounced uncoupling of neuronal activity and blood flow are at a higher risk for the progression or development of diabetes-associated complications. If so, this would allow for the use of functional hyperemia to identify high-risk patients and/or the use of functional hyperemia as a surrogate parameter for disease progression. Further, studies investigating the mechanism of neurovascular breakdown in diabetes are required. 

## 8. Conclusions

Neurovascular coupling is one of the key physiological mechanisms that allow for the adaptation of the retinal vascular system to changing neural function and increased metabolic demand. Although the exact molecular mechanisms behind this functional hyperemic response warrant further investigation, there is general agreement that an impairment of neurovascular coupling is an early event in several ocular and systemic diseases including diabetic retinopathy. Thus, it is reasonable to suggest that an impaired response to increased metabolic demand might trigger further detrimental consequences such as focal ischemia or insufficient removal of metabolic waste products. Further research is needed to develop an accurate model of the regulation of blood flow in the eye to allow for a better understanding of why blood flow is compromised in patients with diabetes. In addition, the observation made in animal models that impaired neurovascular response can be at least partially restored by modulating the messengers within the neurovascular unit opens new perspectives on drug targets for early pharmacological interventions before the full clinical picture of the disease is established. Finally, as the breakdown of neurovascular coupling is an early event in the disease process, impaired functional hyperemia might be used as a surrogate parameter for the identification of high-risk patients as well as for the follow-up of treatment outcomes. However, longitudinal studies are required to further investigate neurovascular coupling during the disease process.
